# How do women who are informed that they are at increased risk of breast cancer appraise their risk? A systematic review of qualitative research

**DOI:** 10.1038/s41416-022-01944-x

**Published:** 2022-08-24

**Authors:** Victoria G. Woof, Anthony Howell, Lorna McWilliams, D. Gareth Evans, David P. French

**Affiliations:** 1 University of Manchester, Oxford Road, Manchester, M13 9PL UK; 2 The Nightingale Centre, Wythenshawe Hospital, Manchester University NHS Foundation Trust, Southmoor Road, Manchester, M23 9QZ UK

**Keywords:** Breast cancer, Risk factors, Disease prevention, Patient education

## Abstract

This review aimed to synthesise qualitative research on how women notified that they are at increased risk of breast cancer view their risk. Five electronic databases were systematically reviewed for qualitative research investigating how women who have received an increased breast cancer risk estimate appraise their risk status. Fourteen records reporting 12 studies were included and critically appraised. Data were thematically synthesised*.* Four analytical themes were generated. Women appraise their risk of breast cancer through comparison with their risk of other familial diseases. Clinically derived risk estimates were understood in relation to pre-conceived risk appraisals, with incongruences met with surprise. Family history is relied upon strongly, with women exploring similarities and differences in attributes between themselves and affected relatives to gauge the likelihood of diagnosis. Women at increased risk reported living under a cloud of inevitability or uncertainty regarding diagnosis, resulting in concerns about risk management. Women hold stable appraisals of their breast cancer risk which appear to be mainly formed through their experiences of breast cancer in the family. Healthcare professionals should explore women’s personal risk appraisals prior to providing clinically derived risk estimates in order to address misconceptions, reduce concerns about inevitability and increase perceived control over risk reduction.

## Background

Since the inception of UK Family History Risk and Prevention Clinics (FHRPCs) in the late 1980s, women with a family history of breast cancer have been able to have their risk of developing the disease estimated. Risk is estimated through combining multiple risk factors, including family history of breast and other relevant cancers (ovary), hormonal and reproductive factors, as well as health related behaviours, i.e. smoking and alcohol consumption. In the UK the National Institute for Health and Care Excellence (NICE) clinical guidelines for familial breast cancer [1] recommend providing women with their 5-year, or 10-year and/or lifetime risk of breast cancer. Advice on risk management, for example additional screening or preventative medication is also provided for those eligible. Such clinics are also available globally, including in the US, Canada and Australia.

As with other areas of medicine where disease risk is provided, risk communication in FHRPCs aims to, (i) reduce anxiety, (ii) motivate risk reducing behaviours and (iii) facilitate informed choices, such as uptake of preventative medication or additional screening [2]. Risk feedback is often provided in numerical formats, i.e. as percentages and probabilities [2, 3]. However, traditional numerical risk feedback has been criticised for being hard to understand and recall [2, 3]. Risk feedback of this kind also typically has little effect on how individuals think about health risks [2]. Understanding risk and forming personal risk appraisals appears to be more complex than merely absorbing the numerical risk information provided in clinical settings [2, 4–7]. Numerical risk information and the use of risk communication strategies produce little effect in comparison to the impact that personal experiences and affective responses can have on subjective risk appraisals [8].

Following a clinically derived risk estimate, women still appraise their risk of breast cancer inaccurately, with genetic counselling only having a modest effect on improving risk perception accuracy [9–12]. Additionally, even when women are able to accurately recall their numerical risk estimate, some believe this estimate does not reflect their personal perceptions, affecting their trust in the estimate provided [13]. These studies indicate that there are other factors which may be influencing women’s tendency to under- or overestimate their risk of breast cancer in spite of receiving a clinically derived risk estimate. A better appreciation of these factors is needed in order to understand how women view their risk and make decisions about risk-related behaviours. However, personal perceptions of risk and how women construct a meaning around their breast cancer risk still remains under investigated in genetic counselling sessions [5, 9, 14, 15]. Instead of assuming that subjective risk appraisals can be easily replaced by the provision of ‘objective’ clinical risk information [13], it has been suggested that specific time should be devoted to exploring personal risk appraisals in clinical consultations [9, 14, 15].

A small body of qualitative literature has explored breast cancer risk appraisals following the provision of a clinically derived risk estimate. However, the extent of discrepancies between a clinically derived risk estimate and women’s personal risk appraisals are still largely unknown, as this literature has not been systematically collated and synthesised. A similar qualitative review [16] has been conducted in this area, however the authors included papers which included participants who had not received a clinically derived risk estimate, as well as those identified as gene mutation carriers. As only 2-5% of breast cancers are attributable to mutations in high penetrance genes [17], the findings presented are unlikely to be representative of the common experience of receiving a clinically derived risk estimate.

A systematic review of qualitative studies where an increased breast cancer risk result has been provided should provide an insight into how those in receipt of a clinically derived risk estimate appraise and understand their risk. Such a review would highlight common misunderstandings or views which could be addressed in clinical settings, improve the accuracy of breast cancer risk appraisals, increase informed decision making, and facilitate better healthcare professional communication. The aim of the present review was to synthesis qualitative research exploring breast cancer risk appraisals in unaffected (those without breast cancer), non-mutation carrier women who have been informed that they are at increased risk of the disease. Specific objectives were to: (i) systematically search and appraise the existing qualitative literature, (ii) use thematic synthesis to develop an understanding of women’s risk appraisals and, (iii) identify areas of misunderstanding to facilitate trust in the risk estimates provided.

## Methods

The protocol for this qualitative systematic review was registered in PROSPERO (CRD42021255930) and is reported in accordance with guidance found in the PRISMA statement [18] (see Supplementary Material 1). The enhancing transparency in reporting the synthesis of qualitative research (ENTREQ) [19] checklist was used in the reporting of this review (see Supplementary Material 2).

### Search strategy

The following electronic databases were searched from 1980 to the present day (July 2021): MEDLINE (ovid host), CINAHL Plus (Ebsco host), APA PsychInfo (ovid host), EMBASE (ovid host), and ProQuest Dissertation and Thesis. These databases were searched from 1980 onwards as the first risk prediction model [20] was being designed and evaluated in this decade. An academic librarian was consulted when developing the search strategy and terms (Supplementary Material 3). Search strategies of similar qualitative and quantitative systematic reviews were also consulted when developing the search strategy [16, 21]. Searches were limited to full-text records available in English. Forward and backward citation searches, as well as first named author searches were performed for all included records. Researchers with expertise in this area were also contacted in order to identify any grey literature and to provide any records which were not retrieved during the database searches.

### Eligibility criteria

Studies were included in the review if they met the following inclusion criteria:

1. Adult women (aged 18+ years) who have received a clinically derived breast cancer risk estimate (5-year, 10-year or lifetime) and classified as above-average (moderate) or high risk in accordance with national guidelines [1] in either a clinic or trial/study setting.
2. Studies which included a focus on moderate and high risk women’s breast cancer risk appraisals, perceptions and views.
3. Mixed sample studies, where samples included women with high penetrance gene mutations or women who have been affected by breast cancer were only included if views from women at increased risk only were reported independently or could be separated.
4. Studies that included risk appraisals regarding other diseases, such as cardiovascular disease and diabetes were only included if views from women at increased risk of breast cancer were reported separately or could be separated.
5. Studies which used any qualitative methodology, including but not restricted to, interviews and focus groups.
6. Studies which used any qualitative analysis method, including but not restricted to, thematic analysis and Interpretive Phenomenological Analysis (IPA).
7. Mixed methods studies, providing that there was a substantial qualitative element illustrated by author interpretation and quotes.

Studies were excluded based on the following criteria:

1. Studies that included participants under the age of 18 years.
2. Studies conducted exclusively with women previously or currently affected by breast cancer.
3. Studies conducted exclusively with women who were confirmed high penetrance gene mutation carriers.
4. Quantitative only studies.

Case studies, opinion pieces, books, conference abstracts and review articles were also excluded from this review.

### Selection process and coding

Search results from each database were downloaded into Endnote where duplicates were highlighted and removed. This Endnote library was then transferred to Rayyan (online systematic review software) [22] in order to complete the screening process. The first author (VGW) screened all titles and abstracts and a second reviewer (AH) screened 30% (*K* = 700) of these (96% agreement). The first author (VGW) then read the full text of all potential records which appeared to meet the eligibility criteria. The second reviewer (AH) read 100% (*K* = 191) of these full text records. Regular discussions were held between VGW and AH to establish disagreements and resolve any issues in order to come to an agreement. A third reviewer (DPF) was consulted when an agreement could not be reached.

### Quality appraisal

A modified version of the Critical Appraisal Skills Programme (CASP) tool [23] was used to assess quality of the qualitative records included. The modified CASP tool was chosen in order to assess the rigour of the records including quality appraisal of the ontological and epistemological underpinnings of the research. Acknowledgement of the researcher’s ontological and epistemological stance and research design choices is considered a marker of quality and transparency in qualitative research and should be included in publications [24]. Similarly, the modified version of the CASP tool includes the response ‘somewhat’ to highlight where the CASP criterion is partially addressed in records.

VGW appraised all included records. Five records reporting three studies (3 published manuscripts and 2 PhD theses (1 thesis included 2 studies that had been published)) were appraised independently, as the level of information and data provided differed between each record. A second reviewer (DPF) appraised two of the included records (*K* = 2; 14%). Differences between reviewers were discussed and resolved. All records that met inclusion criteria were included in the review regardless of quality appraisal results.

### Data extraction

Data was extracted using an excel file, with descriptive data on the included records presented in Table 1. Thomas and Harden’s guidance [25] for thematic synthesis was followed when extracting data from the included records for data synthesis. Therefore, data included within the ‘results’ or ‘findings’ was extracted from the included records, including author’s interpretations and direct quotes from participants.

**Table 1:** Characteristics of the included records.

Author (year)	Country	Participants	Data collection method	Analysis method
Altschuler and Somkin, [38]	USA	28 women (aged 40–79 years) on chemoprevention medication and 23 women (aged 50–80+ years) who declined participation/chemoprevention medication from the STAR trial. All women were eligible for chemoprevention and were at increased risk according to their Gail scores (1.66 5-year risk and above)	In-depth semi-structured interviews	Grounded Theory
Anderson et al., [27]	USA	13 low-income African American (11) and Latina (2) women (aged 18–69 years) from a federally qualified health centre (FQHC) at increased risk of breast cancer using the Breast Cancer Risk Screening (BRS) tool (which incorporates a modified version of the Gail model)	In-depth structured interviews	Conventional content analysis
Appleton et al., [28]	UK	25 women (aged 27–51 years) with a family history of breast cancer. All received genetic counselling and were under clinical surveillance for 2 or more years. Lifetime risk for all women was between 20 and 40%	Telephone focus groups and feedback questionnaire	Vaughn et al.’s (1996) modified of guidelines for analysing focus groups
Bennett et al., [29]	UK	30 women (mean age 49.5 years) from the TRACE study found to be at intermediate risk of breast cancer using the Claus model 6 years prior to interviewing	In-depth semi-structured interviews	Thematic analysis
Gunn et al., [30]	USA	30 women (mean age 50.9 years) at increased risk of breast cancer using the Gail model. Women were recruited from the National Surgical Adjuvant Breast and Bowel Project (NSABP/NRG Oncology Decision-Making Project (DMP)-1) study	In-depth semi-structured interviews	Kleinman et al.’s explanatory model domains and grounded thematic approach
Gunn et al., [31]	USA	30 women (mean age 50.9 years) at increased risk of breast cancer using the Gail model who had been counselled about chemoprevention medication. Women were recruited from the National Surgical Adjuvant Breast and Bowel Project (NSABP/NRG Oncology Decision-Making Project (DMP)-1) study	In-depth semi-structured interviews	Kleinman et al.’s explanatory model domains and constant comparative approach
Gunn, [32] (thesis describing Gunn et al., [30] and Gunn et al., [31])	USA	30 women (mean age 50.9 years) at increased risk of breast cancer using the Gail model. Women were recruited from the National Surgical Adjuvant Breast and Bowel Project (NSABP/NRG Oncology Decision-Making Project (DMP)-1) study	In-depth semi-structured interviews	Kleinman et al.’s explanatory model domains and grounded thematic approach
Hallowell et al., [33]	UK	46 women (aged 22–59 years) who attended a Cancer Family History Clinic in Cambridge for hereditary breast or ovarian cancer	Semi-structured telephone interviews, face-to-face semi-structured interviews and postal questionnaires	Grounded Theory
Holmberg et al., [39]	USA	40 (aged 44–74 years) women eligible to take part in the Study of Tamoxifen and Raloxifene (STAR) study. 20 women agreed to participate and 20 who refused. All women were at increased risk and eligible for chemoprevention according to Gail model scores	In-depth narrative interviews	Person-by-person analysis and thematic analysis
Phelps et al., [40]	UK	157 women undergoing genetic assessment for familial breast-ovarian cancer risk. 97 women (40 under 40 years, 55 over 40 years) provided free-text responses upon referral to the clinic, 60 (21 under 40 years, 38 over 40 years) provided responses prior to receiving their risk result (average, moderate or high) and 36 women provided responses following their risk result. Of the women providing responses following notification of risk (*n* = 36), 10 were at high risk and 22 at moderate risk of breast–ovarian cancer	Free-text questionnaires	Thematic Analysis
Robertson, [34]	Canada	20 low-, medium- and high-risk pre-menopausal women (aged between 30 and 50 years) from a Breast Health Clinic situated in a teaching hospital in Canada	Focus group and in-depth semi-structured interviews	'*Standard qualitative data analysis*’, briefly mentions thematic analysis
Schroeder et al., [35]	Canada	9 women (aged 25–58 years) with at least a 20% lifetime risk of breast cancer recruited from a hereditary breast and ovarian cancer clinic in Western Canada	Conversational interviews	van Manen’s hermeneutic phenomenological approach
Schroeder, [36] (thesis describing Schroeder et al., study)	Canada	9 women (aged 25–58 years) with at least a 20% lifetime risk of breast cancer recruited from a hereditary breast and ovarian cancer clinic in Western Canada	Conversational interviews	van Manen’s hermeneutic phenomenological approach
Scott et al., [37]	UK	58 men and women at low, moderate and high risk of breast, ovarian, colorectal and other cancers were recruited from a UK cancer genetics service. 17 women (age not provided) in the sample were at moderate or high risk of breast cancer	In-depth semi-structured interviews	Grounded Theory

### Data synthesis

Thomas and Harden’s [25] thematic synthesis method was employed. This approach was chosen in order to remain faithful to the ideas presented in the included studies but also to apply our own interpretations and explanations to produce a greater understanding of breast cancer risk appraisals. As a theoretically free method, the present thematic synthesis was informed by critical realism. Here the researcher appreciates that there is an external reality accessible to all, however individuals can only share so much of this reality, as experiences of this reality are subjective and contextualised [26]. Therefore, the researcher views the social world presented in the records of this synthesis as accounts of both the participants’ and researchers’ interpretations of the experience of being at increased risk of breast cancer within a particular social and cultural context.

Data were analysed in NVivo version 12. Data analysis began with inductive line-by-line coding. This form of coding facilitated the translation of concepts between data sets. First, participant quotes were coded, then authors’ narratives. Records assessed as being higher in quality were coded first, with these codes applied to medium quality records. Codes generated from the high and medium quality records were applied to those assessed as being lower in quality. No new codes were created at this stage.

The subsequent development of descriptive themes was achieved by combining codes together based on their conceptual meanings and relatedness. Groupings were given a theme name to capture their content. The final analysis stage involved developing analytical themes where the researcher (VGW) engaged in interpretation of the descriptive themes by going beyond what was said in the original studies. This was done by considered the conceptual links between descriptive themes in order to develop new insights and concepts. The final analytical themes generated were discussed multiple times within the research team (VGW, DPF and AH) before a final thematic structure was agreed.

## Results

Searches of the electronic databases yielded 3499 results (see Supplementary Material/Fig. 1, PRISMA 2020 flow chart). From the screening process fourteen records, describing twelve studies (with three studies described in five records) were included (see Table 1).

### Characteristics of the included studies

All included records were published between 1998 and 2019 (see Table 1). Of the 14 records included, six were conducted in the US, five in the UK and three in Canada. The primary aim of eleven records was to explore how women experience, view, perceive, describe, make sense of, conceive of or adjust to being at increased risk of breast cancer [27–37]. Two records focused on women’s decision making for taking preventative medication; interview questions also focused on perceptions of being at increased risk of breast cancer [38, 39]. One record’s primary aim was to explore women’s experiences of the genetic risk assessment process; thoughts on risk were explored following the notification of results [40].

An increased risk of breast cancer was defined by the Gail risk assessment model in six of the included records. A lifetime risk of above 20% defined an increased risk of breast cancer in three records. The Claus risk assessment model was used in one record. Four records identified women at increased risk of breast cancer due to being in follow-up or following their attendance at either family history clinics, cancer genetic services or breast health clinics. How risk was calculated is not provided in these four records.

### Quality appraisal results

The quality of the included records ranged from high to low. Only six records provided information on the theoretical underpinnings of the research, with the remaining eight either providing modest information or no information at all regarding their ontological and epistemological stance. Details regarding the influence of the researcher when undertaking qualitative research, including their impact on the design and study results was either modestly or poorly acknowledged, apart from in two records. The quality of the records were mixed in regards to the rigour of the data analysis. Seven records were identified as significantly rigorous, whilst four were somewhat rigorous. The remaining records provided limited information in order to assess the rigour of the data analysis. Three of the records included were considered to be of low quality, based on the rigour of the analysis process, as well as whether there was a clear statement of the findings. These records were not excluded from the analysis but instead the data extracted was used to support the codes and themes produced from the analysis of the high and medium quality records (see Supplementary Material 4 for CASP results).

### Thematic synthesis results

Thematic synthesis of the 14 included records resulted in seven descriptive themes, organised into four analytical themes: (i) *breast cancer risk is not the only priority, (ii) congruency between personal risk appraisals and clinical estimates, (iii) comparative predictors of breast cancer risk and (iv) living under a breast cancer cloud* (see Supplementary Material 5 for thematic map). Quotes from women are indicated by quotation marks and italics, with author narratives indicated by italics only.

#### Breast cancer risk is not the only priority

The risk of breast cancer and the worry associated with the development of the disease was not always prioritised by the women in the studies reviewed. Instead, breast cancer risk was weighed up against the risks of other diseases present in the family. For example, many women discussed their personal risk of breast cancer in relation to other common disease risks, for example risk of cardiovascular disease, stroke or diabetes. Breast cancer risk was not seen in isolation of other disease risks, with many women describing breast cancer risk *as one health risk among many* [31]. Some created a hierarchy of disease risk, where the concern for the development of a given disease was stronger the more prevalent the disease was in the family. For example, in a US study exploring low-income women’s views on risk assessment [27], it was noted that breast cancer did not worry some women, as *other health issues more prevalent in their family, such as diabetes and hypertension, were perceived as more immediate threats*. A *hierarchy of worry* [38] was also associated with grading personal risk of disease, where it was suggested that *women can only worry about so much* [38]. In particular, there was one instance where the possibility of developing breast cancer was viewed more favourably than experiencing a stroke:

*“I don’t know which I would prefer cancer or the stroke. I think probably cancer because a stroke, I mean that just renders you, you know, not able to function pretty much in a lot of cases.”* [30]

Women also considered the severity of their breast cancer risk when deciding whether or not to engage in preventative behaviours. Many identified that health-related behaviours, such as diet or quitting smoking were beneficial for the prevention of all diseases, not just breast cancer. Some women considered the uncertainty of breast cancer development in relation to changes to their lifestyles, with striving for a normal life considered a higher priority:

*“I’m not eating any more blueberries, cutting out alcohol completely, or stopping my birth control pill. I’m trying to live a normal life because I don’t know if this is actually gonna happen for me or not.”* [35]

Specifically the severity of their breast cancer risk was often used to determine whether or not to take up the offer of preventative medications. Again for some the use of preventative medications were considered in relation to medications already taken for current health issues. One women described her use of medication for migraines, making the distinction between symptoms already present compared to a risk that cannot be seen or which may not manifest into disease:

*“[A drug] is something I take for migraines because it allows me to function when I get my migraine headaches. I take one and it curbs the headache enough that I can continue to work or not be impaired…and the risks [migraine v. breast cancer] just aren’t the same.”* [31]

#### Congruency between personal risk appraisals and clinical estimates

Prior to receiving a clinically derived risk estimate, it is evident that women have pre-existing ideas about their breast cancer risk and expectations for what the estimate will reveal. For some women the clinical risk estimate did not correlate with their breast cancer risk appraisals, with clinical estimates either being too low or higher than expected. Those who believed their risk to be lower than the clinical estimate provided acknowledged their health-behaviours and lack of a ‘strong’ family history to defend their personal risk appraisals. However, some women did accept their clinical risk estimates, but did not fully integrate this information into their own personal risk appraisals as, *while they understood intellectually that they were at heightened risk, such calculated risk did not cause them worry* [38], indicating that perhaps personal risk appraisals are preferred over clinical risk estimates when expectations are not met. For women who were informed that their risk was lower than expected, clinical risk estimates often came as a surprise, with some women dubious about whether the information can be trusted or relied upon:

“*I thought I’d be higher. In fact, I still thought I’d be higher, yet because she’s [her mother] postmenopausal they explained that the risk wouldn’t be that high….But these are just statistics and I can’t really go by statistics, you know? All because it says I may not be, you know, at average risk or you’re a little higher than average risk; that doesn’t mean anything […] so I can’t really say I really trust the number that they gave me, you know?”* [39]

Often these women did not feel reassured by the estimate provided, or reassurance was short lived given that their personal risk appraisals were not confirmed. In contrast, women who suspected themselves to be at increased risk and had this confirmed described being unsurprised, as the estimate was *not new information…given their knowledge of their family history and the role that this plays in breast cancer* [27]. For these women the clinical risk estimate provided validation for their personal risk appraisals, confirming what they had ‘known’ for a long time:

*“My moderate risk is what I expected, I don’t feel worried about my risk assessment. I have known for over twenty years I have an increased risk…”* [40]

Compared to those where expectations were not met, these women were satisfied with their risk results and appeared not to question the accuracy of the risk assessment, nor did they find the results particularly worrying.

#### Comparative predictors of breast cancer risk

Women appreciated that breast cancer is caused by multiple risk factors including, ageing, health behaviours and environmental factors. In line with the previous theme, the most cited of these causes was family history. The amount of experience of familial breast cancer women had helped them make sense of their own breast cancer risk and influenced their personal risk appraisals. Women with a strong family history of breast cancer used this information to surmise that they were at high risk:

*“Well because of family history. I am bound to be at a bigger risk. I mean possibly I am not carrying the gene, but I know I am more at risk maybe than I would normally be*.*”* [37]

The significance that women attributed to family history was also used to make sense of clinical risk estimates, with a lack of family history in some cases used to judge their level of risk, irrespective of the contributions of other risk factors:

*“…on my father’s side of the family, even though he had colon cancer, which was not what he died of, and his sister didn’t have breast cancer, my cousins have not had breast cancer. So, you know, I’d like to think that maybe I’m not really as much at risk as the paperwork said I was.”* [39]

However, family history for a minority of women was not seen as the defining factor influencing their risk. Instead these women placed greater value on maintaining positive health behaviours, describing that *the familial link overstated their own risk* [38]. These women further acknowledged that although an important risk factor, many breast cancers do not have links to familial predisposition. Nevertheless, the majority of women used their familial experiences of breast cancer to make sense of their own risk and their likelihood of developing the disease. Specifically to make sense of their risk women compared their attributes and characteristics to that of their affected relatives. These included comparing physical attributes, such as the size of their breasts, as well as behavioural characteristics, for example lifestyle choices and diet. Here, women identified differences between themselves and their affected relatives to provide reassurance that differences were sufficient to reduce any concerns about developing breast cancer:

*“I understand the whole cell dividing thing, but I have a very different lifestyle than my mother did. And not that she brought that on herself, but she had many diseases, and because she didn’t take care of herself, and she was the total opposite of what I am.”* [31]

One the other hand, the identification of similarities also caused women to fear that they would follow the same path to disease as their affected relatives, “*I feel like it’s just, it’s going to happen because I have the same breasts as my mom*” [36]. A particularly significant factor when drawing comparisons between the self and affected relatives was age. Women used the age of disease onset in affected relatives as a milestone when appraising their risk, with this period of time describe in a UK study [28] *as a psychological cut-off date*. Women made a distinction between how they thought about their breast cancer risk when approaching the age of onset and when they had moved passed this age. Women described approaching the age where their relatives were diagnosed as an anxious time, with one woman describing age 45, the age at which here mother was diagnosed as “*my scary age*” [35]. Passing the age of onset without a diagnosis brought relief, which a US study [38] acknowledged as providing *a diminished sense of risk because they had passed the age at which a close family member had developed breast cancer*.

#### Living under a breast cancer cloud

For most women, the thought of developing breast cancer was something that could not be ignored considering their increased risk status and family history, with one women describing her breast cancer worry as a *“cancer backpack”* [36]. Some held fatalistic views or felt *doomed by the presumed eventuality of* developing breast cancer [38], considering a diagnosis as an inevitability:

*“Everyone in my family has cancer. You know, I lost members from cancer… I feel ‘oh nothing’s going to happen to me. That’s just them. It’s going to skip me.’ No, nothing skips… Somewhere along the line it’s going to invade my body and I know that.”* [31]

For some of these women the view that breast cancer was an inevitability was paired with their appraisals of whether breast cancer risk or the development of breast cancer could be controlled. The strength of women’s family history and their lack of control over this was drawn upon here, as well as thoughts regarding the randomness of breast cancer, “*it’s just something that just, it happens and you only have so much control*” [30]. Some of these women were dubious about preventative advice, namely health behaviour advice, drawing on examples of women who had lived healthy lives but still developed breast cancer. However, this was not the view of all women, as those who believed that breast cancer risk could be reduced by living healthily felt less fatalistic and more in control of their risk:

*“… all these things [exercise, healthy diet, stopping smoking] help you to feel you are in control of the situation rather than the situation is in control of your life so I think it is very important to do things, positive things, to make you feel you are doing all you can, you know, not to have it (breast cancer).”* [28]

Other women described living in a perpetual state of uncertainty. For these women not knowing when or if they would ever receive a diagnosis of breast cancer was anxiety inducing:

*“…it’s the not knowing that drives you crazy, [rather] than the knowing. If you know, then you can deal with it a lot better than not knowing… they can’t guarantee me [anything].”* [31]

In one study, this uncertainty was described as *dangerous* as women may cause themselves undue distress worrying about something that may never happen [28]. Alternatively, there were some women who did not dwell on their breast cancer risk or the uncertainty of development, citing their positive health behaviours, lack of family history or the prevalence of other familial diseases as reasons not to worry about their breast cancer risk too much.

## Discussion

This systematic review of qualitative research revealed that women who receive a clinically derived breast cancer risk estimate appraise this estimate in relation to the threat of other diseases, with family history of disease influencing a hierarchy of worry. When receiving a clinically derived risk estimate, women appear to have pre-existing expectations, with results rejected if expectations are not met or if personal risk appraisals are not validated. In contrast, when clinical risk estimates confirm expectations, results were unsurprising. Although women acknowledged that there are multiple risk factors associated with breast cancer, the majority cited family history as the main predictor. Women rely on making comparisons between the self and affected relatives to make sense of their own personal risk of breast cancer, with some women holding misunderstandings about breast cancer indicators. The majority of women either viewed the diagnosis of breast cancer as inevitable or lived with constant uncertainty, affecting whether they viewed breast cancer risk as something that could be reduced or controlled.

### Relevance to existing literature

This review has highlighted that women’s breast cancer risk appraisals are emotionally laden, drawing on experiential memories of cancer in affected relatives. This is further evidence that individuals do not appear to think about health risks in probabilistic terms [2, 16], indicating that numerical risk estimates cannot overcome such strong emotional sources of risk appraisals [5, 8, 13]. Additionally, these strong personal risk appraisals appear to guide expectations regarding clinically derived risk estimates, shown in other cancer types [6, 7]. These findings indicate that risk communication may not always resonate or fit with pre-existing personal risk appraisals [2] causing some to distrust or reject clinical risk estimates. This review has also emphasised the apparent incongruence between the way healthcare-professionals and women appraise breast cancer risk, highlighting common misconceptions that women hold, as well as the significant role family history plays in determining subjective appraisals of risk. Furthermore, how breast cancer risk is appraised in relation to risk of other diseases has also been identified. The present review has specifically highlighted that family history of other known diseases and risks associated with their development can be prioritised over a risk of breast cancer. This novel finding differs from a similar qualitative review where the threat of breast cancer is likely to be viewed more significantly among confirmed mutation carriers and women affected by breast cancer [16].

Comparative indicators, such as comparing attributes and behaviours between the self and affected friend or relative has been cited previously in the literature as helping individuals make sense of their disease risk [7, 16, 41–43]. Specifically the findings of this review indicate that breast size, health behaviour comparisons and age of disease onset in affected relatives are key indicators used to determine personal threat of breast cancer. From this review it was found that misconceptions such as comparing breast size appear to lead to feelings of certainty about development or provide false reassurance in the case of passing the age of disease onset. Similar findings regarding the age of disease onset have also been found in those affected by breast cancer and confirmed mutation carriers [16]. As with the present review, it was found that approaching the age of disease onset of the affected relative was a significant stage in women’s lives, with passing this age described as a relief. As numerical risk estimates have been criticised for being hard to understand [3], it is unsurprising that probabilistic risk communication interventions have had little impact on risk appraisals, as the effect of vivid comparative indicators and mental images, which can so easily be brought to mind, are overlooked [44].

### Strengths and limitations

This qualitative systematic review was conducted in line with the PRISMA and ENTREQ guidelines and associated protocol published on PROSPERO. This review provides an in-depth exploration how unaffected non-mutation carrier women who have received a breast cancer risk estimate appraise and experience their risk. Therefore by explicitly focusing on women’s appraisals of their breast cancer risk a deeper understanding has been achieved and nuances of receiving an increased breast cancer risk uncovered. As a result it has become clear that there is incongruence between how healthcare professionals and women think about breast cancer risk. This review has emphasised these issues, indicating that current risk communication practices need to change in order to incorporate an understanding of subjective risk appraisals.

Limitations of the present review were that we included studies which were written in English only which may have resulted in the exclusion of views from different cultures. The quality of included studies was mixed, with the majority appraised as medium quality and as three poor. In addition, the primary aim of some of the included studies did not centre exclusively on how women at increased risk appraise their risk [see refs. [27, 35, 36]]. Therefore more quality in-depth research is needed with women who receive breast cancer risk estimates in order to explicitly explore how they appraise and experience their risk. This will be particularly important if risk estimation is introduced into population-based breast screening programmes [see refs. [45–47]].

### Clinical implications

It can be inferred from the present findings that for some women breast cancer risk is not seen in isolation of other disease risks, especially if multiple diseases run in the family. In the FHRPCs HCPs are focused on providing women with the most salient information about their risk, so that accurate risk appraisals can be made to encourage changes in risk-related behaviours, as well as to aid informed decision making about preventative action. However, in this same consultation women may be appraising their breast cancer risk against that of other diseases present in the family, drawing on their experiences to assess where their priorities for monitoring risk should lie. This may mean that for some breast cancer risk is not an immediate priority, potentially affecting their motivation to engage in preventative behaviours. For these women a multiple disease prevention programme (MDPP) whereby preventative behaviours (i.e. increasing physical activity and maintaining a healthy diet) associated with reducing the risk of multiple diseases may be more appropriate than a prevention programme linked to breast cancer risk alone [48, 49]. However when compared to a Breast Cancer Prevention Programme (BCPP) a MDPP showed no significant improvements in physical activity and diet, indicating that perhaps a MDPP would only be appropriate in a minority of cases [48, 49] as this review suggests.

Family history is a significant component of women’s breast cancer risk appraisals, although this is less important on a population basis. Specifically women look for comparisons between the self and affected relative, i.e. comparing breast size to gage the likelihood of diagnose. It would be beneficially for HCPs to explore misunderstandings such as this in clinically settings to facilitate more accurate risk appraisals. One method to improve understanding and dispel myths about the link between breast size and breast cancer risk might be to discuss the issue of breast density. Breast density notification in consultations where images from mammography are shown to illustrate the differing levels of fatty tissue to fibro-glandular tissue may improve women’s understanding, moving them away from inaccurate ideas about breast size. For example, providing medical images in the communication of disease risk has been found to motivate risk reducing behaviours [50]. Therefore, providing visual images from a woman’s own mammography appointment and comparing these images to that of others where breast density differs could make a meaningful impression [50], increasing the likelihood of this information being incorporated into pre-existing risk appraisals.

Findings from this review also highlight incongruences between ‘objective’ clinically derived risk estimates and women’s personal breast cancer appraisals. In some cases this misalignment leads to distrust in the clinical estimates provided. A distrust of this kind may cause women to feel dubious about risk reducing behaviours and prevention advice. Similarly, this review found that the majority of women who received an increased breast cancer risk estimate feel that diagnosis is inevitable, leading to thoughts that risk cannot be controlled or a diagnosis prevented. It is important therefore for the HCP to acknowledge how women appraise their risk and identify where the clinical risk estimate may differ from subjective understandings of risk. Identifying where incongruences lie may improve the accuracy of these risk appraisals, as well as instil trust in the estimate and prevention advice provided. One way of initiating such conversations could be to employ risk perception instruments such as the Tripartite model of risk perception [51], which captures cognitive processes such as, perceived susceptibility (deliberative) to the disease, affective responses and experiential factors (heuristic-based judgements). Results from such tools could be used to provoke tailored discussions around personal risk appraisals.

### Further research

This comprehensive review of the literature indicates that there is a discrepancy between how healthcare professionals communicate breast cancer risk estimates and how women appraise breast cancer risk. Future research should investigate how common these discrepancies are and whether these vary between healthcare professionals communicating risk results to women. Additionally, an investigation into the implications of healthcare professional-patient incongruences, for example regarding uptake of preventive options to reduce breast cancer risk would also be helpful.

Since the publication of the included studies breast cancer risk prediction models have improved, with the inclusion of new strong independent risk factors, breast density and a polygenic risk score (PRS) [52–56]. It is evident from this review that family history is a significant contributing factor when women form an appraisal of risk. With this in mind, future research should focus on how and whether women integrate these new risk factors into their pre-existing risk appraisals and whether these appraisals alter as a result. An exploration of this kind would highlight how new risk factors are understood, as well as how they should be communicated in order for women to form accurate risk appraisals.

Risk prediction models which include a PRS and breast density are likely to be formally introduced into clinical settings in the near future. It is therefore expected that women under follow-up at FHRPCs will receive updated breast cancer risk estimates. Updated estimates could result in women experiencing a change to their risk and risk management, for example their entitlement to more frequent screening. It is currently unknown how women react to a change in their breast cancer risk. However from the present review it is evident that women question clinically derived risk estimates when they do not validate existing risk appraisals. Future research should explore how and if risk appraisals are adapted when women receive an updated breast cancer risk estimate, including whether there is any psychological impact. An exploration as to whether a change in breast cancer risk and entitlement to preventative management effects the perceived trustworthiness of risk estimates would also be beneficial.

## Conclusions

Clinically derived risk estimates are evaluated against personal appraisals of risk, with women shocked and dubious about results when results misalign with pre-existing risk appraisals. Those at increased risk appear to view the development of breast cancer as an inevitability or live in uncertainty, causing women to question whether breast cancer can be prevented or risk reduced. The findings of this review have practical value for HCPs by increasing their awareness of how women think about breast cancer risk, which may result in more effective and meaningful communication in clinical settings.

## Supplementary information

Supplementary Material

## Data Availability

Data extracted and synthesised are available from the corresponding author upon request.
