# Supplementary material for: How do women who are informed that they are at increased risk of breast cancer appraise their risk? A systematic review of qualitative research
Source: Br J Cancer. 2022 Aug 24;127(11):1916–24. doi: 10.1038/s41416-022-01944-x (PMC9681857; doi:10.1038/s41416-022-01944-x)
Supplement: Supplementary file 1 — Supplementary Material [file 41416_2022_1944_MOESM1_ESM.docx]

**Supplementary material 1: PRISMA checklist**

| **Section and Topic** | **Item #** | **Checklist item** | **Location where item is reported** |
| --- | --- | --- | --- |
| **TITLE** | | |  |
| Title | 1 | Identify the report as a systematic review. | 1,2,4 |
| **ABSTRACT** | | |  |
| Abstract | 2 | See the PRISMA 2020 for Abstracts checklist. | 2 |
| **INTRODUCTION** | | |  |
| Rationale | 3 | Describe the rationale for the review in the context of existing knowledge. | 3,4 |
| Objectives | 4 | Provide an explicit statement of the objective(s) or question(s) the review addresses. | 3,4 |
| **METHODS** | | |  |
| Eligibility criteria | 5 | Specify the inclusion and exclusion criteria for the review and how studies were grouped for the syntheses. | 4,5,6,8 |
| Information sources | 6 | Specify all databases, registers, websites, organisations, reference lists and other sources searched or consulted to identify studies. Specify the date when each source was last searched or consulted. | 4 |
| Search strategy | 7 | Present the full search strategies for all databases, registers and websites, including any filters and limits used. | Supplementary file |
| Selection process | 8 | Specify the methods used to decide whether a study met the inclusion criteria of the review, including how many reviewers screened each record and each report retrieved, whether they worked independently, and if applicable, details of automation tools used in the process. | 4,5,6,8 |
| Data collection process | 9 | Specify the methods used to collect data from reports, including how many reviewers collected data from each report, whether they worked independently, any processes for obtaining or confirming data from study investigators, and if applicable, details of automation tools used in the process. | 4,5,6,8 |
| Data items | 10a | List and define all outcomes for which data were sought. Specify whether all results that were compatible with each outcome domain in each study were sought (e.g. for all measures, time points, analyses), and if not, the methods used to decide which results to collect. | 4,5,6,8 |
|  | 10b | List and define all other variables for which data were sought (e.g. participant and intervention characteristics, funding sources). Describe any assumptions made about any missing or unclear information. | 4,5,6,8 |
| Study risk of bias assessment | 11 | Specify the methods used to assess risk of bias in the included studies, including details of the tool(s) used, how many reviewers assessed each study and whether they worked independently, and if applicable, details of automation tools used in the process. | 6,9, supplementary file |
| Effect measures | 12 | Specify for each outcome the effect measure(s) (e.g. risk ratio, mean difference) used in the synthesis or presentation of results. | n/a |
| Synthesis methods | 13a | Describe the processes used to decide which studies were eligible for each synthesis (e.g. tabulating the study intervention characteristics and comparing against the planned groups for each synthesis (item #5)). | Table 1 |
|  | 13b | Describe any methods required to prepare the data for presentation or synthesis, such as handling of missing summary statistics, or data conversions. | n/a |
|  | 13c | Describe any methods used to tabulate or visually display results of individual studies and syntheses. | n/a |
|  | 13d | Describe any methods used to synthesize results and provide a rationale for the choice(s). If meta-analysis was performed, describe the model(s), method(s) to identify the presence and extent of statistical heterogeneity, and software package(s) used. | 8,9 |
|  | 13e | Describe any methods used to explore possible causes of heterogeneity among study results (e.g. subgroup analysis, meta-regression). | n/a |
|  | 13f | Describe any sensitivity analyses conducted to assess robustness of the synthesized results. | n/a |
| Reporting bias assessment | 14 | Describe any methods used to assess risk of bias due to missing results in a synthesis (arising from reporting biases). | n/a |
| Certainty assessment | 15 | Describe any methods used to assess certainty (or confidence) in the body of evidence for an outcome. | n/a |
| **RESULTS** | | |  |
| Study selection | 16a | Describe the results of the search and selection process, from the number of records identified in the search to the number of studies included in the review, ideally using a flow diagram. | 8, PRISMA flow diagram |
|  | 16b | Cite studies that might appear to meet the inclusion criteria, but which were excluded, and explain why they were excluded. | PRISMA diagram |
| Study characteristics | 17 | Cite each included study and present its characteristics. | Table 1 |
| Risk of bias in studies | 18 | Present assessments of risk of bias for each included study. | 9, supplementary file |
| Results of individual studies | 19 | For all outcomes, present, for each study: (a) summary statistics for each group (where appropriate) and (b) an effect estimate and its precision (e.g. confidence/credible interval), ideally using structured tables or plots. | 9, supplementary file |
| Results of syntheses | 20a | For each synthesis, briefly summarise the characteristics and risk of bias among contributing studies. | 9, supplementary file |
|  | 20b | Present results of all statistical syntheses conducted. If meta-analysis was done, present for each the summary estimate and its precision (e.g. confidence/credible interval) and measures of statistical heterogeneity. If comparing groups, describe the direction of the effect. | 8-14 |
|  | 20c | Present results of all investigations of possible causes of heterogeneity among study results. | n/a |
|  | 20d | Present results of all sensitivity analyses conducted to assess the robustness of the synthesized results. | n/a |
| Reporting biases | 21 | Present assessments of risk of bias due to missing results (arising from reporting biases) for each synthesis assessed. | n/a |
| Certainty of evidence | 22 | Present assessments of certainty (or confidence) in the body of evidence for each outcome assessed. | n/a |
| **DISCUSSION** | | |  |
| Discussion | 23a | Provide a general interpretation of the results in the context of other evidence. | 14-18 |
|  | 23b | Discuss any limitations of the evidence included in the review. | 15 |
|  | 23c | Discuss any limitations of the review processes used. | 15 |
|  | 23d | Discuss implications of the results for practice, policy, and future research. | 14-18 |
| **OTHER INFORMATION** | | |  |
| Registration and protocol | 24a | Provide registration information for the review, including register name and registration number, or state that the review was not registered. | 4 |
|  | 24b | Indicate where the review protocol can be accessed, or state that a protocol was not prepared. | 4 |
|  | 24c | Describe and explain any amendments to information provided at registration or in the protocol. | See PROSPERO |
| Support | 25 | Describe sources of financial or non-financial support for the review, and the role of the funders or sponsors in the review. | 1 |
| Competing interests | 26 | Declare any competing interests of review authors. | 1 |
| Availability of data, code and other materials | 27 | Report which of the following are publicly available and where they can be found: template data collection forms; data extracted from included studies; data used for all analyses; analytic code; any other materials used in the review. | n/a |

*From:*  Page MJ, McKenzie JE, Bossuyt PM, Boutron I, Hoffmann TC, Mulrow CD, et al. The PRISMA 2020 statement: an updated guideline for reporting systematic reviews. BMJ 2021;372:n71. doi: 10.1136/bmj.n71

For more information, visit: <http://www.prisma-statement.org/>

**Supplementary materials 2**

ENTREQ checklist (Enhancing transparency in reporting the synthesis of qualitative research) *

| No. Item | Guide questions/description | Reported |
| --- | --- | --- |
|  |  | on Page # |
| 1. Aim | State the research question the synthesis addresses |  |
|  |  | 2,4 |
| 2. Synthesis Methodology | Identify the synthesis methodology or theoretical framework which underpins | 8 |
|  | the synthesis, and describe the rationale for choice of methodology (e.g. |  |
|  | meta-ethnography, thematic synthesis, critical interpretive synthesis, |  |
|  | grounded theory synthesis, realist synthesis, meta-aggregation, meta-study, |  |
|  | framework synthesis) |  |
|  |  |  |
| 3. Approach to strategy | Indicate whether the search was pre-planned (comprehensive search | 4-8 |
|  | strategies to seek all available studies) or iterative (to seek all available |  |
|  | concepts until they theoretical saturation is achieved) |  |
|  |  |  |
| 4. Inclusion criteria | Specify the inclusion/exclusion criteria (e.g. in terms of population, language, | 4-5 |
|  | year limits, type of publication, study type) |  |
|  |  |  |
| 5. Data sources | Describe the information sources used (e.g. electronic databases (MEDLINE, | 4-5 |
|  | EMBASE, CINAHL, psycINFO), grey literature databases (digital thesis, policy |  |
|  | reports), relevant organisational websites, experts, information specialists, |  |
|  | generic web searches (Google Scholar) hand searching, reference lists) and |  |
|  | when the searches conducted; provide the rationale for using the data sources |  |
|  |  |  |
| 6. Electronic Search strategy | Describe the literature search (e.g. provide electronic search strategies with | 4-6 |
|  | population terms, clinical or health topic terms, experiential or social |  |
|  | phenomena related terms, filters for qualitative research, and search limits) |  |
|  |  |  |
| 7. Study screening methods | Describe the process of study screening and sifting (e.g. title, abstract and full | 4-6 |
|  | text review, number of independent reviewers who screened studies) |  |
|  |  |  |
| 8. Study Characteristics | Present the characteristics of the included studies (e.g. year of publication, | Table 1 |
|  | country, population, number of participants, data collection, methodology, |  |
|  | analysis, research questions) |  |
|  |  |  |
| 9. Study selection results | Identify the number of studies screened and provide reasons for study |  |
|  | exclusion (e,g, for comprehensive searching, provide numbers of studies | 8, PRISMA flow diagram |
|  | screened and reasons for exclusion indicated in a figure/flowchart; for |  |
|  | iterative searching describe reasons for study exclusion and inclusion based on |  |
|  | modifications to the research question and/or contribution to theory |  |
|  | development) |  |
|  |  |  |
| 10. Rationale for appraisal | Describe the rationale and approach used to appraise the included studies or | 6,9 |
|  | selected findings (e.g. assessment of conduct (validity and robustness), |  |
|  | assessment of reporting (transparency), assessment of content and utility of |  |
|  | the findings) |  |
|  |  |  |
| 11. Appraisal items | State the tools, frameworks and criteria used to appraise the studies or | 6 |
|  | selected findings (e.g. Existing tools: CASP, QARI, COREQ, Mays and Pope [25]; |  |
|  | reviewer developed tools; describe the domains assessed: research team, |  |
|  | study design, data analysis and interpretations, reporting) |  |
|  |  |  |
| 12. Appraisal process | Indicate whether the appraisal was conducted independently by more than | 6 |
|  | one reviewer and if consensus was required |  |
|  |  |  |
| 13. Appraisal results | Present results of the quality assessment and indicate which articles, if any, | 9, supplementary file |
|  | were weighted/excluded based on the assessment and give the rationale |  |
|  |  |  |
| 14. Data extraction | Indicate which sections of the primary studies were analysed and how were | 8 |
|  | the data extracted from the primary studies? (e.g. all text under the headings  “results /conclusions” were extracted electronically and entered into a |  |
|  | computer software) |  |
|  |  |  |
| 15. Software | State the computer software used, if any | 6,8 |
|  |  |  |
| 16. Number of reviewers | Identify who was involved in coding and analysis | 8 |
|  |  |  |
|  |  |  |
| 17. Coding | Describe the process for coding of data (e.g. line by line coding to search for | 8 |
|  | concepts) |  |
|  |  |  |
| 18. Study comparison | Describe how were comparisons made within and across studies (e.g. | 8 |
|  | subsequent studies were coded into pre-existing concepts, and new concepts |  |
|  | were created when deemed necessary) |  |
|  |  |  |
| 19. Derivation of Themes | Explain whether the process of deriving the themes or constructs was | 8 |
|  | inductive or deductive |  |
|  |  |  |
| 20. Quotations | Provide quotations from the primary studies to illustrate themes/constructs, | 9-14 |
|  | and identify whether the quotations were participant quotations of the |  |
|  | author’s interpretation |  |
|  |  |  |
| 21. Synthesis Output | Present rich, compelling and useful results that go beyond a summary of the | 9-14 |
|  | primary studies (e.g. new interpretation, models of evidence, conceptual |  |
|  | models, analytical framework, development of a new theory or construct) |  |
|  |  |  |

- Reference: Tong A, Flemming K, McInnes E, Oliver SA, Craig J. Enhancing transparency in reporting the synthesis of qualitative research: ENTREQ. BMC Medical Research Methodology 2012, 12:181.

**Supplementary materials 3**

Search terms and search results by database

**Ovid Medline R (1946 - July Week 2 2021) – search strategy**

1. Breast cancer.mp. (251,652 hits)
2. Breast Neoplasms/(MeSH term) (300,071 hits)
3. Breast carcinoma.mp. (23,329 hits)
4. 1 or 2 or 3 (353, 568 hits)
5. (risk or elevated risk or high risk or higher risk or moderate risk or above average risk or increased risk or risk assessment* or risk estimate* or at risk or familial risk or heredit* or predispos* or susceptib*).mp. [mp=title, abstract, original title, name of substance word, subject heading word, floating sub-heading word, keyword heading word, organism supplementary concept word, protocol supplementary concept word, rare disease supplementary concept word, unique identifier, synonyms] (2,941,128 hits)
6. (view* or opinion* or perce* or belie* or experience* or attitude* (MeSH term) or appraisal* or perspective* or feeling* or thought*).mp. [mp=title, abstract, original title, name of substance word, subject heading word, floating sub-heading word, keyword heading word, organism supplementary concept word, protocol supplementary concept word, rare disease supplementary concept word, unique identifier, synonyms] (3,357,081 hits)
7. (risk perception* or risk understand* or perceived risk or risk belie* or attitude* to risk or threat perception* or threat* or perceived threat* or perceived severit* or perceived vulnerability).mp. [mp=title, abstract, original title, name of substance word, subject heading word, floating sub-heading word, keyword heading word, organism supplementary concept word, protocol supplementary concept word, rare disease supplementary concept word, unique identifier, synonyms] (205, 988 hits)
8. (risk adj3 perception*).mp. [mp=title, abstract, original title, name of substance word, subject heading word, floating sub-heading word, keyword heading word, organism supplementary concept word, protocol supplementary concept word, rare disease supplementary concept word, unique identifier, synonyms] (8,582 hits)
9. (risk adj3 understand*).mp. [mp=title, abstract, original title, name of substance word, subject heading word, floating sub-heading word, keyword heading word, organism supplementary concept word, protocol supplementary concept word, rare disease supplementary concept word, unique identifier, synonyms] (6,524 hits)
10. 7 or 8 or 9 (214,275 hits)
11. 5 and 6 and 10 (26,494 hits)
12. 4 and 11 (954 hits)
13. (interview* or focus group* or survey* or questionnaire* or observ* or discourse analysis or content analysis or interpretive phenomenological analysis or IPA or thematic analysis or narrative analysis or conversation analysis or grounded theory or qualitative analysis or qualitative or mixed method*).mp. [mp=title, abstract, original title, name of substance word, subject heading word, floating sub-heading word, keyword heading word, organism supplementary concept word, protocol supplementary concept word, rare disease supplementary concept word, unique identifier, synonyms] (4,579,510 hits)
14. 12 and 13 (624 hits) (623 when limited to 1980-2021)

(search from 1980-2021, qual or mixed methods only)

**EBSCOhost CINAHL Plus – search strategy**

1. Breast cancer (98,631 hits)
2. (MH “Breast Neoplasms”) (87,750 hits)
3. Breast carcinoma (74,559 hits)
4. 1 or 2 or 3 (109,373 hits)
5. Risk or elevated risk or high risk or higher risk or moderate risk or above average risk or increased risk or risk assessment* or risk estimate* or at risk (1,016,888 hits)
6. familial risk or heredit* or predispos* or susceptib* (100,290 hits)
7. 5 or 6 (1,077,234 hits)
8. view* or opinion* or perce* or belie* or experience* or attitude* or appraisal* or perspective* or feeling* or thought* (1,350,218 hits)
9. risk perception* or risk understand* or perceived risk or risk belie* or attitude* or threat perception* or threat* or perceived threat* or perceived severit* or perceived vulnerability (435,187 hits)
10. (MH “attitude to risk”) (2,030 hits)
11. 9 or 10 (435,187 hits)
12. 7 and 8 and 11 (62, 404 hits)
13. 12 and 4 (1,989 hits)
14. interview* or focus group* or survey* or questionnaire* or observ* or discourse analysis or content analysis or interpretive phenomenological analysis or IPA or thematic analysis or narrative analysis or conversation analysis or grounded theory or qualitative analysis or qualitative or mixed method* (1,453,993 hits)
15. 13 and 14 (1,427 hits)

(search from 1981-2021 (database begins at 1981), qual or mixed methods only)

**Ovid APA PsycInfo (1806 to July Week 2 2021) – search strategy**

1. Breast cancer.mp. (13,292 hits)
2. exp Breast Neoplasms/ (MeSH term) (10,443 hits)
3. Breast carcinoma.mp. (103 hits)
4. 1 or 2 or 3 (13,786 hits)
5. (risk or elevated risk or high risk or higher risk or moderate risk or above average risk or increased risk or risk assessment* or risk estimate* or at risk or familial risk or heredit* or predispos* or susceptib*).mp. [mp=title, abstract, heading word, table of contents, key concepts, original title, tests & measures, mesh] (472,162 hits)
6. (view* or opinion* or perce* or belie* or experience* or attitude* or appraisal* or perspective* or feeling* or thought*).mp. [mp=title, abstract, heading word, table of contents, key concepts, original title, tests & measures, mesh] (2,194,438 hits)
7. (risk perception* or risk understand* or perceived risk or risk belie* or attitude* to risk or threat perception* or threat* or perceived threat* or perceived severit* or perceived vulnerability).mp. [mp=title, abstract, original title, name of substance word, subject heading word, floating sub-heading word, keyword heading word, organism supplementary concept word, protocol supplementary concept word, rare disease supplementary concept word, unique identifier, synonyms] (85,837hits)
8. (risk adj3 perception*).mp. [mp=title, abstract, heading word, table of contents, key concepts, original title, tests & measures, mesh] (11,729 hits)
9. (risk adj3 understand*).mp. [mp=title, abstract, heading word, table of contents, key concepts, original title, tests & measures, mesh] (3,240 hits)
10. 7 or 8 or 9 (90,214 hits)
11. 5 and 6 and 10 (22,531 hits)
12. 4 and 11 (574 hits)
13. (interview* or focus group* or survey* or questionnaire* or observ* or discourse analysis or content analysis or interpretive phenomenological analysis or IPA or thematic analysis or narrative analysis or conversation analysis or grounded theory or qualitative analysis or qualitative or mixed method*).mp. [mp=title, abstract, heading word, table of contents, key concepts, original title, tests & measures, mesh] (1,548,272 hits)
14. 12 and 13 (378 hits) (376 when limited to 1980-2021)

(search from 1980-2021, qual or mixed methods only)

**Ovid EMBASE (1974 to 2021, July 16) – search strategy**

1. Breast cancer.mp. (546,139 hits)
2. Breast cancer/ (MeSH term) (387,036 hits)
3. Breast neoplasm.mp. (2,058 hits)
4. Breast tumor/ (MeSH term) (85,158 hits)
5. Breast carcinoma.mp. (84,680 hits)
6. Breast carcinoma/ (MeSH term) (71,382 hits)
7. 1 or 2 or 3 or 4 or 5 or 6 (619,001 hits)
8. (risk or elevated risk or high risk or higher risk or moderate risk or above average risk or increased risk or risk assessment* or risk estimate* or at risk or familial risk or heredit* or predispos* or susceptib*).mp. [mp=title, abstract, heading word, drug trade name, original title, device manufacturer, drug manufacturer, device trade name, keyword, floating subheading word, candidate term word] (5,053,752 hits)
9. (view* or opinion* or perce* or belie* or experience* or attitude* or appraisal* or perspective* or feeling* or thought*).mp. [mp=title, abstract, heading word, drug trade name, original title, device manufacturer, drug manufacturer, device trade name, keyword, floating subheading word, candidate term word] (5,066,644 hits)
10. (risk perception* or risk understand* or perceived risk or risk belie* or attitude* to risk or threat perception* or threat* or perceived threat* or perceived severit* or perceived vulnerability).mp. [mp=title, abstract, heading word, drug trade name, original title, device manufacturer, drug manufacturer, device trade name, keyword, floating subheading word, candidate term word] (324,805 hits)
11. (risk adj3 perception*).mp. [mp=title, abstract, heading word, drug trade name, original title, device manufacturer, drug manufacturer, device trade name, keyword, floating subheading word, candidate term word] (12,970 hits)
12. (risk adj3 understand*).mp. [mp=title, abstract, heading word, drug trade name, original title, device manufacturer, drug manufacturer, device trade name, keyword, floating subheading word, candidate term word] (11,184 hits)
13. 10 or 11 or 12 (338,356 hits)
14. 8 and 9 and 13 (43,578 hits)
15. 7 and 14 (1,621 hits)
16. (interview* or focus group* or survey* or questionnaire* or observ* or discourse analysis or content analysis or interpretive phenomenological analysis or IPA or thematic analysis or narrative analysis or conversation analysis or grounded theory or qualitative analysis or qualitative or mixed method*).mp. [mp=title, abstract, heading word, drug trade name, original title, device manufacturer, drug manufacturer, device trade name, keyword, floating subheading word, candidate term word] (7,444,421 hits)
17. 15 and 16 (1001 hits) (997 when limited to 1980-2021)

**ProQuest Dissertation and Theses – search strategy 21.07.2021**

1. noft(Breast cancer)
2. AND noft(elevated risk OR high risk OR higher risk OR moderate risk OR above average risk OR increased risk OR risk assessment* OR risk estimate*)
3. AND noft(risk perception* OR risk appraisal* OR risk understand* OR perceived risk)
4. AND noft(qualitative OR mixed method*)
5. 76 hits (1980-2021)

noft = anywhere except full text (like title and abstract)

**Supplementary material 4: CASP, quality appraisal results for included records**

| Authors (year) | Modified CASP question | | | | | | | | | |
| --- | --- | --- | --- | --- | --- | --- | --- | --- | --- | --- |
|  | 1. Was there a clear statement of the aims of the research? | 2. Is a qualitative methodology appropriate? | 3. Was the research design appropriate to address the aims of the research? | 4. Are the study's theoretical underpinnings (e.g. ontological and epistemological assumptions; guiding theoretical framework(s)) clear, consistent and conceptually coherent? | 5. Was the recruitment strategy appropriate to the aims of the research? | 6. Was the data collected in a way that addressed the research issue? | 7. Has the relationship between researcher and participants been adequately considered? | 8. Have ethical issues been taken into consideration? | 9. Was the data analysis sufficiently rigorous? | 11. Is there a clear statement of findings? |
| Altschuler & Somkin (2005) | Yes | Yes | Yes | Somewhat | Yes | Yes | No | Somewhat | Yes | Somewhat |
| Anderson et al (2018) | Somewhat | Yes | Somewhat | No | Somewhat | Somewhat | No | Can’t tell | Somewhat | No |
| Appleton et al (2000) | Yes | Yes | Yes | No | Yes | Yes | No | Yes | Somewhat | Yes |
| Bennett et al (2010) | Yes | Yes | Yes | No | Yes | Yes | No | Somewhat | Can’t tell | No |
| Gunn et al (2019a) | Yes | Yes | Yes | Yes | Yes | Yes | No | Yes | Yes | Yes |
| Gunn et al (2019b) | Yes | Yes | Yes | Yes | Yes | Yes | No | Yes | Yes | Yes |
| Gunn (2015) | Yes | Yes | Yes | Yes | Yes | Yes | No | Yes | Yes | Yes |
| Hallowell et al (1998) | Yes | Yes | Yes | No | Yes | Somewhat | Somewhat | Can’t tell | No | Somewhat |
| Holmberg et al (2015) | Yes | Yes | Yes | Somewhat | Yes | Yes | Somewhat | Can’t tell | Yes | Yes |
| Phelps et al (2007) | Yes | Yes | Somewhat | No | Somewhat | Can’t tell | Yes | Yes | Somewhat | Yes |
| Robertson (2000) | Yes | Yes | Yes | Somewhat | Yes | Somewhat | No | No | Can’t tell | Somewhat |
| Schroeder et al (2017) | Yes | Yes | Yes | Yes | Yes | Yes | Somewhat | Yes | Yes | Yes |
| Schroeder (2016) | Yes | Yes | Yes | Yes | Yes | Yes | Yes | Yes | Yes | Yes |
| Scott et al (2005) | Yes | Yes | Yes | Somewhat | Can’t tell | Somewhat | No | Can’t tell | Somewhat | Somewhat |

**Supplementary material 5: Thematic map**

| **Analytical themes** | | | | | | | |
| --- | --- | --- | --- | --- | --- | --- | --- |
| Breast cancer risk is not the only priority | | Congruency between personal risk appraisals and clinical estimates | | Comparative predictors of breast cancer risk | | Living under the breast cancer cloud | |
| **Descriptive themes** | | | | | | | |
| Breast cancer is one risk among many | Severity of risk vs preventative action | Preconceived expectations of risk | Indicators of breast cancer and risk | The strength and influence of family history | Inevitability of breast cancer development | | Breast cancer development is uncertain |
| **Codes** | | | | | | | |
| *- There are risks to everything*  *- Hierarchy of disease risk*  *-Every women has a risk of BC*  *- BC risk vs current illness and comorbidities*  *- BC risk and other disease in the family* | *- Striving for a normal life – changes in lifestyle behaviours*  *- Lifestyle prevention attributable to all of disease*  *- Already does everything possible regarding a healthy lifestyle*  *- Lifestyle changes vs the uncertainty of development*  *- Preventative medication use vs risk of BC* | *- Calculated risk does not meet expectations*  *- Satisfied with calculated risk estimate*  *- Calculated risk confirmed expectations*  *- Unconvinced by calculated risk estimate*  *- Accepted but does not believe estimate* | *- Affected relatives age of onset vs own age*  *- Threat intensifies at same age, threat depletes after passing the age*  *- Making sense of risk by comparing physical attributes of affected women*  *- Self vs affected relative, attributes do not correlate*  *- Comparing lifestyle habits with affected women* | *- Risk appraisals associated with level of FH*  *- High risk associated with genetic links*  *- BC is a hereditary disease*  *- FH does not point to a high risk of BC*  *- FH influence for seeking risk assessment* | *- BC is an immediate threat*  *- BC is always at the forefront*  *- BC is going to happen, just a matter of when*  *- Fatalistic about BC development*  *- BC is inevitable because level of FH*  *- BC cannot be controlled/prevented*  *(Not the view of all)*  *- Cynical about lifestyle advice (But some do believe in a healthy lifestyle* to reduce risk)  *- Lifestyle changes and BC risk reduction not always associated* | | - Does *not dwell on BC development*  *- BC could happen but might not*  *- BC development is random*  *- Anxiety of the uncertainty of BC development* |

**Figure 1. PRISMA 2020 flow chart**

**Identification of studies via databases and registers**

**Identification of studies via other methods**

Records removed *before screening*:

Duplicate records removed (n=1,222)

Records identified from:

Citation searching (n = 14)

etc.

Records identified from:

Databases (n=3,499; CINAHL Plus n=1,427, EMBASE n=997, Medline n=623, PsycINFO n=376, ProQuest Dissertations & Theses Global n=76)

**Identification**

Records excluded

(n=2,096)

Records screened

(n=2,277)

Records not retrieved

(n = 0)

Records sought for retrieval

(n = 14)

Records not retrieved

(n = 4)

Records sought for retrieval

(n=181)

**Screening**

Records excluded:

Conference proceeding (n = 24)

Thesis abstract only (n = 1)

Full text not in English (n = 4)

Quantitative outcomes only (n = 43)

Ineligible population (women had not received a formal clinical risk estimate or no evidence of a formal clinical risk estimate provided) (n = 31)

Ineligible population (BRCA positive women or affected by breast cancer) (n = 19)

Ineligible population (low risk sample) (n = 1)

Mixed sample (unable to isolate relevant material) (n = 15)

Incomplete risk estimate (PRS only) (n = 2)

Unclear population (n = 6)

Insufficient qualitative data (n = 5)

No breast cancer risk appraisal specific data (n = 15)

Commentary piece (n = 4)

Systematic review (n = 1)

Narrative review (n = 1)

Records assessed for eligibility

(n = 177)

Records excluded:

Ineligible population (BRCA positive women or affected by breast cancer) (n = 2)

Ineligible population (women had not received a formal clinical risk estimate or no evidence of a formal clinical risk estimate provided (n = 1)

Mixed sample (unable to isolate relevant material) (n = 3)

Records assessed for eligibility

(n = 14)

n=6

Total studies included in present review (n = 12 reported in 14 records (3 studies reported in 5 records, 3 publications and 2 theses))

Studies to be included in qualitative synthesis (n = 12 reported in 14 records)

n=8

**Included**
